# Three novel compound heterozygous *IL12RB1* mutations in Chinese patients with Mendelian susceptibility to mycobacterial disease

**DOI:** 10.1371/journal.pone.0215648

**Published:** 2019-04-18

**Authors:** Xiaopei Zhou, Weimin Jia, Zhengyi Ni, Ali Wang, Zhenxing Liu, Meiqi Hou, Mi Zhou, Zhongwen Tang, Dazhi Zhang, Lei Li, Tiantian Han, Yang Tan, Geng Luo, Jiarui Wang, Yanling Wu, Xianqin Zhang

**Affiliations:** 1 Key Laboratory of Molecular Biophysics of the Ministry of Education, College of Life Science and Technology and Center for Human Genome Research, Huazhong University of Science and Technology, Wuhan, Hubei, China; 2 Wuhan Jinyintan Hospital, Wuhan, Hubei, China; German Cancer Research Center (DKFZ), GERMANY

## Abstract

Mendelian Susceptibility to Mycobacterial Diseases (MSMD) is a primary immunodeficiency disease (PID) characterized by variable susceptibility to weakly virulent mycobacteria (Bacille Calmette-Guerin, BCG) and various intramacrophagic bacteria, fungi, parasites. Mycobacterial disease generally begins in childhood, more rarely during adolescence and adulthood. The pathogenesis of MSMD is the inherited impaired production of interferon gamma (IFN-γ) or inadequate response to it. Autosomal recessive *IL12RB1 *deficiency is the most common genetic etiology of MSMD. Here we identified three novel compound heterozygous mutations in *IL12RB1* gene (c.635G>A, c.765delG; c.632G>C, c.847C>T; c.64G>A, c.1673insGAGCTTCCTGAG) in three Chinese families with MSMD.

## Introduction

Mendelian susceptibility to mycobacterial disease (MSMD) is a rare inherited disease characterized by selective vulnerability to infections by weakly virulent mycobacteria (Bacillus Calmette-Guerin Vaccine, BCG) and other intramacrophagic bacteria (listeriosis, nocardiosis, klebsiellosis), fungi (candidiasis, histoplasmosis, paracoccidioidomycosis, coccidioidomycosis) and parasites (leishmaniasis, toxoplasmosis) [1]. MSMD mostly begins in childhood, and has various clinical manifestations, ranging from regional to disseminated infections with one or more mycobacterial species that may or may not recur [1]. The first disease gene of MSMD was identified in 1996, which was caused by bi-allelic null mutations of *IFNGR1* [2]. Up to now, eleven MSMD-causing genes, including nine autosomal (*IFNGR1*, *IFNGR2*, *STAT1*, *IL12B*, *IL12RB1*, *ISG15*, *IRF8*, *RORC* and *TYK2* and two X-linked (*NEMO*, and *CYBB*) genes have been identified [1, 3, 4]. IFN-γ is important for killing and controlling mycobacterial infections [5–6]. Most of these MSMD-disease genes mutations lead to either insufficient production of IFN-gamma (γ) or inadequate response to it [7].

Autosomal recessive IL-12Rβ1 deficiency is the most common genetic etiology of MSMD [1, 6, 8]. IL-12Rβ1 is a receptor chain that not only combines with IL-12Rβ2 to form the receptor for IL12 but also combines with IL23R to form the receptor for IL23 [9]. NK and T cells from patients with MSMD who carry the *IL12RB1* mutations do not respond to IL-12 and produce low levels of IFN-γ [10]. In this study, we identified three different novel compound heterozygous mutations in *IL12RB1* gene in three MSMD patients.

## Materials and methods

### Ethics statement

Three unrelated Chinese MSMD patients from Wuhan jinyintan hospital were included in this study. The project was approved by the ethics committee of Huazhong University of Science and Technology. All participants in the study agreed with informed consent to participate in the investigation.

### Case report

**Fig 1:**
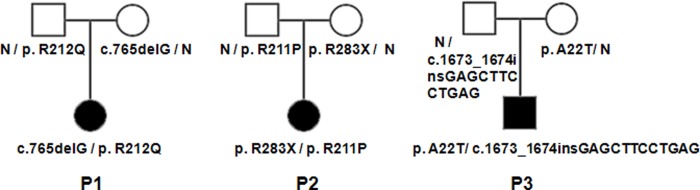
Pedigrees of MSMD families. The patients with MSMD indicated with P1, P2, P3. All the patients of MSMD carry the compound heterozygous mutations in *IL12RB1* gene.

Patient 1 is a girl who received BCG vaccine when she was 4 months old, then a lump of finger size began to appear under her left armpit. When they came to the Wuhan jinyintan hospital, the lump had appeared for nearly four months. Blood routine examination displayed the number of leukocytes were increased. The patient was given debridement and treatment with anti-inflammatory drug of cephalosporins as well as isoniazid (INH). Patient 2 is a girl who received BCG vaccine when she was 3 months old. After one month, her parents found a lump about 3.0× 2.0cm in her left armpit. The patient was treated with anti-infection and external application of Chinese Medicine. Patient 3 is a boy, who received BCG vaccine, a big lump in the left armpit were found by his parents when he was 8 months old. The lump’s size like a pigeon egg. Puncture showed tuberculosis in lymph nodes and acid-fast stain is positive in these three patients, the three patients also received other test and were diagnosed with Mendelian susceptibility to mycobacterial disease (MSMD) in Wuhan Jingyintan hospital. Pedigree of the three families were shown in Fig 1.

### Mutation analysis

Peripheral blood was obtained from all patients and their parents after obtaining informed consent. Genomic DNA was extracted from blood samples using standard methods. All exons and intron–exon boundaries of MSMD disease genes were sequenced: *IFNGR1*, *IFNGR2*, *STAT1*, *IL12B*, *IL12RB1*, *ISG15*, *IRF8*, *RORC*, *NEMO*, *CYBB* and *TYK2*. DNA sequencing analysis was performed using the BigDye Terminator Cycle Sequencing v3.1 kit and an ABI PRISM 3500 Genetic Analyzer (Applied Biosystems, Foster City, CA).

### Plasmids construction

The human *IL12RB1* cDNA were synthesized in The Beijing Genomics Institute (BGI). Mut Express II Fast Mutagenesis Kit V2 of Vazyme Biotech Co.,Ltd were used to create expression vectors for pEGFP-C1-*IL12RB1* wild-type (WT), A22T, R212Q, 765delG, R211P, R283X fusion proteins. Mutations were confirmed by sanger DNA sequencing.

### Confocal and fluorescence microscopy

Hela cells were plated on chamber slide flaskettes (Nalge Nunc, Naperville, IL) and were transiently transfected with expression vectors as described above. Twenty-four hours later cells were fixed in 4% formaldehyde in phosphate-buffered saline (PBS), pH 7.4. The nucleus of cells were stained with DAPI and then visualized by confocal microscopy (×100 oil immersion lens).

### Extraction and analysis of total proteins

HEK293T cells were plated onto 6-well plate and transfected with wild type or mutant IL-12Rβ1 expression vectors using Lipofectamine 2000 (Invitrogen). Cells were used for experiments 48h after transfection.

Western blot analysis of whole cell lysate were performed to check the expression level of wild and mutant IL-12Rβ1 protein. Total proteins were separated by 10% SDS/PAGE gels, and transferred to polyvinylidene difluoride (PVDF) membranes. The membranes were incubated with GFP and GAPDH antibodies. The signals were detected with Bio-RAD ChemiDoc XRS+ imaging system. (Bio-RAD, USA).

### Extraction and analysis of membrane proteins

HEK293T cells were cultured on 10cm cell culture dish one day and transfected with pEGFP-C1, pEGFP-C1-WT and pEGFP-C1-A22T, R211P, R212Q *IL12RB1* using Lipofectamine 2000 (Invitrogen). 48h later after transfection, HEK293T cells were lysed and membrane proteins were extracted with biotin labeling.

Cells of each tissue culture dishes were incubated with 1 mg/ml Sulfo-NHS-SS-Biotin (Pierce, USA) in PBS(2ml) for 30 min. Excess biotin was quenched using 200 mM glycine for 2 min. Then, cells were collected and lysed with cold lysis buffer (50 mM Tris-HCl (pH 7.5), 1% NP-40 substitute (Sigma), 150 mM NaCl, 2 mM EDTA, protease inhibitor) in ice for 40 min, vortexed every five minutes. Then, the mixture were centrifuged at 10,000g and membrane proteins stayed in the supernatant. Finally, the supernatant were incubated with Pierce NeutrAvidin Agarose (Thermo Fisher) at room temperature for one hour and membrane proteins were obtained after the Pierce NeutrAvidin Agarose were incubated with Sample Buffer (62.5 mM Tris-HCl (pH 6.8), 1% SDS, 10% glycerol, 50mM DTT) for three hours.

Membrane proteins were separated by 10% SDS/PAGE gels, and transferred to polyvinylidene difluoride (PVDF) membranes. The membranes were incubated with Na^+^/K^+^- ATPase and GFP antibodies. The signals were detected with Bio-RAD ChemiDoc XRS+ imaging system. (Bio-RAD, USA).

## Results

### DNA sequencing result of patient 1

**Fig 2:**
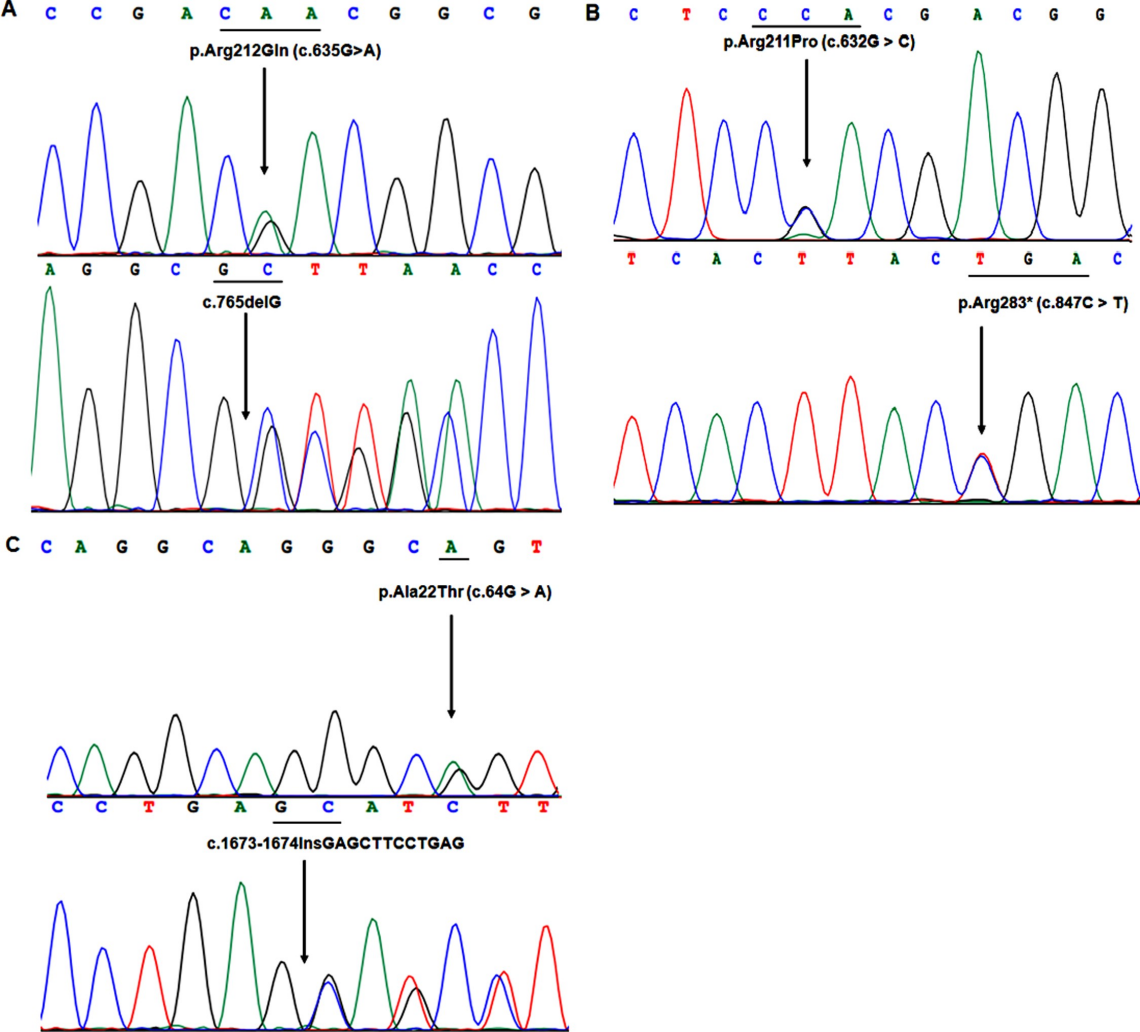
Sanger sequencing results of *IL12RB1* gene in three MSMD patients. **2A)**. DNA Sequencing results show that P1 carry two heterozygous mutations (c.635G > A and c.765del G) **2B)**. Direct DNA sequencing show that P2 carry two heterozygous mutations (c.632G > and c.847C > T). **2C)**. DNA sequence analysis indicated P3 carry two heterozygous mutations (c.64G > A and c.1673insGAGCTTCCTGAG).

Direct DNA sequencing of all exons and exon-intron boundaries of all known disease genes of MSMD, the *IL12RB1* gene in Patient 1 carry two heterozygous mutations, a missense mutations (c.635G > A, R212Q) and a deletion mutation (c.765delG) (Fig 2A). The missense mutation c.635G > A (R212Q) leads the substitution of arginine to glutamine at 212th amino acids. The deletion mutation is caused by deletion of the third base (G) of 255th genetic codon. The mother of the patient carry the c.635G > A mutation, while her father carry the c.765delG mutation. Both of her parents did not suffer from MSMD. Furthermore, 100 unrelated normal controls who received BCG vaccine and without MSMD did not carry the two mutations, this mutation also not reported in other database (1000 Genomes Project and NHLBI Exome Sequencing Project).

### DNA sequencing result of patient 2

DNA sequence analysis identified a novel compound heterozygous mutation in patient 2, a missense mutation and a nonsense mutation in the *IL12RB1* gene, c.632G > C (R211P) and c.847C > T (R283X) (Fig 2B). Both of the two mutations are also heterozygous. The missense mutation c.632G > C (R211P) leads the substitution of arginine to proline at position 211th amino acids. The nonsense mutation c.847C > T (R283X) is caused by the codon, which leads arginine mutated to terminator codon. The two mutations are not single nucleotide polymorphism (SNP) in the Ensembl database. In addition, 100 unrelated normal controls (who received BCG) had not the two mutations. After sequencing to *IL12RB1* of her parents, we found that the father of the patient carry the mutation c.632G > C (R211P) and her mother carry the c.847C > T (R283X) mutation. The patient’s parents have no symptoms of MSMD.

### DNA sequencing result of patient 3

DNA sequencing analysis of all exons and exon-intron boundaries of the *IL12RB1* gene, we identified the patient carried a compound heterozygous mutation, c.64G > A (A22T), c.1673insGAGCTTCCTGAG (Fig 2C). This missense mutation leads the substitution of codon alanine to threonine at amino acid 22th. The framshit insertion mutation is located in exon 14 and occurred between the second base and the third base in the 558th amino acids. These two mutations are not single nucleotide polymorphism (SNP) in the Ensembl database. DNA sequencing analysis of the *IL12RB1* gene from the DNA of the parents revealed that mutation c.64G > A was of maternal origin, while mutation c.1673insGAGCTTCCTGAG was of paternal origin. Similarly, his parents did not suffer from MSMD and 100 unrelated normal controls who received BCG vaccine and without MSMD did not carry the two mutations.

### Subcellular localization of IL-12Rβ1 in Hela cells

**Fig 3:**
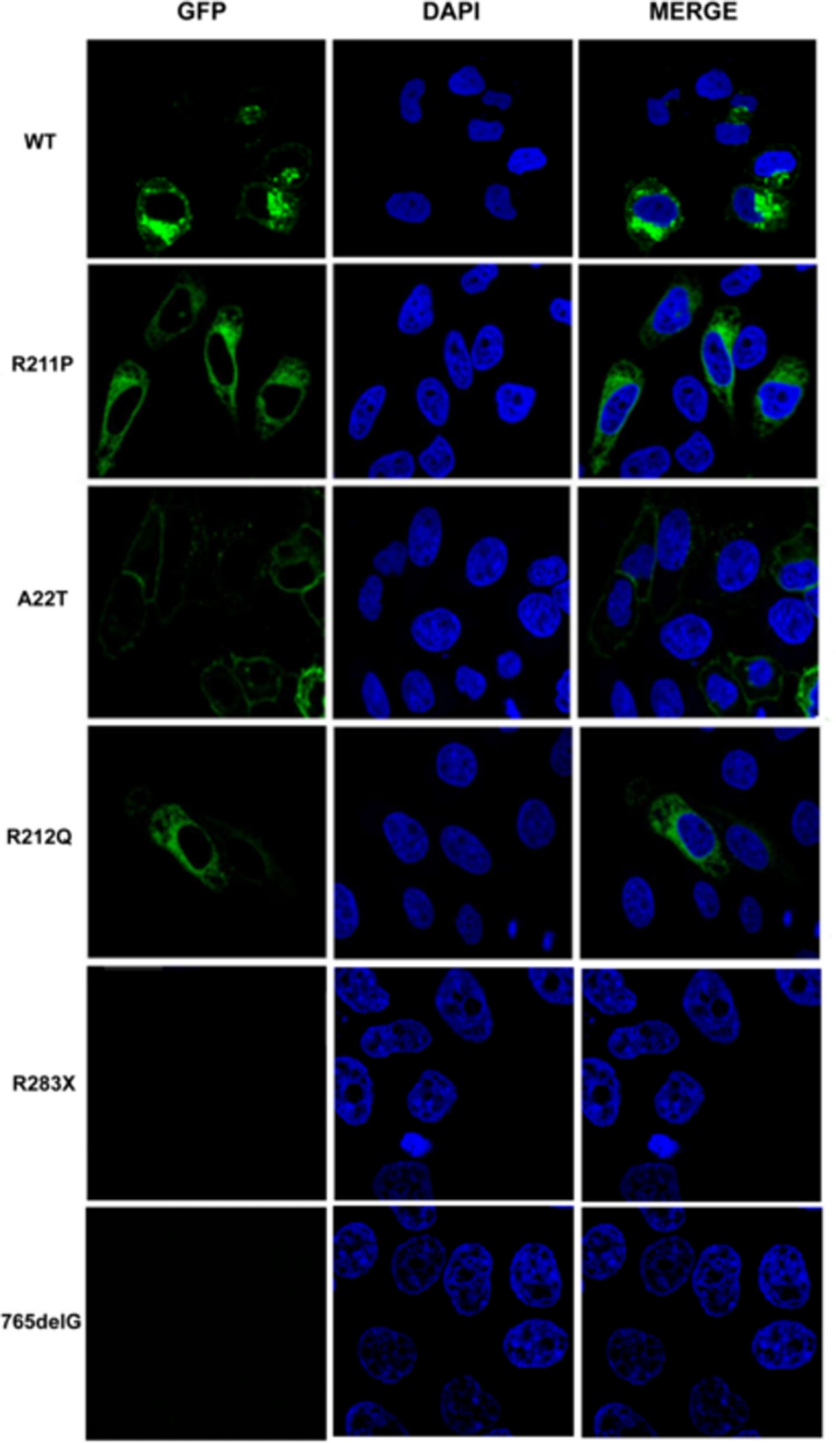
Subcellular localization of wild type and mutant IL-12Rβ1 proteins (A22T, R211P, R212Q, R283X, 765delG) which fused to the green fluorescent protein. Plasmids were transfected into Hela cells. Twenty-four hours later after transfection, cells were fixed in 4% formaldehyde and the nuclei of cells and then stained with DAPI (blue color). Obviously, wild type IL-12Rβ1 proteins were detected mainly localized in cytoplasm and cell membrane, while A22T, R211P, R212Q, IL-12Rβ1 protein were reduced in the cell membrane and disseminated in cytoplasm. Moreover, R283X and 765delG mutant IL-12Rβ1 proteins were not detected. Fluorescence was visualized by confocal microscopy.

IL-12Rβ1 is a receptor in cell membrane, we then check the subcellular distribution of the mutant IL-12Rβ1 protein. Hela cells were transfected with GFP tagged wild and mutant IL-12Rβ1 expression vector pEGFP-C1-*IL12RB1* using Lipofectamine 2000 (Invitrogen) to observed the localization of wild and mutant IL-12Rβ1 protein in the cell. The localization of the proteins was visualized by confocal microscopy (Fig 3). Wild-type IL-12Rβ1 protein were predominantly located in the cytoplasm and cell membrane. By contrast, A22T, R211P, R212Q IL-12Rβ1 proteins were disseminated in cytoplasmic vesicles, there are few mutant protein localized in the cell membrane. R283X and 765delG IL-12Rβ1 mutant proteins do not express as nonsense-mediated mRNA decay (NMD).

### Expression level of IL-12Rβ1 in the HEK293T cells

**Fig 4:**
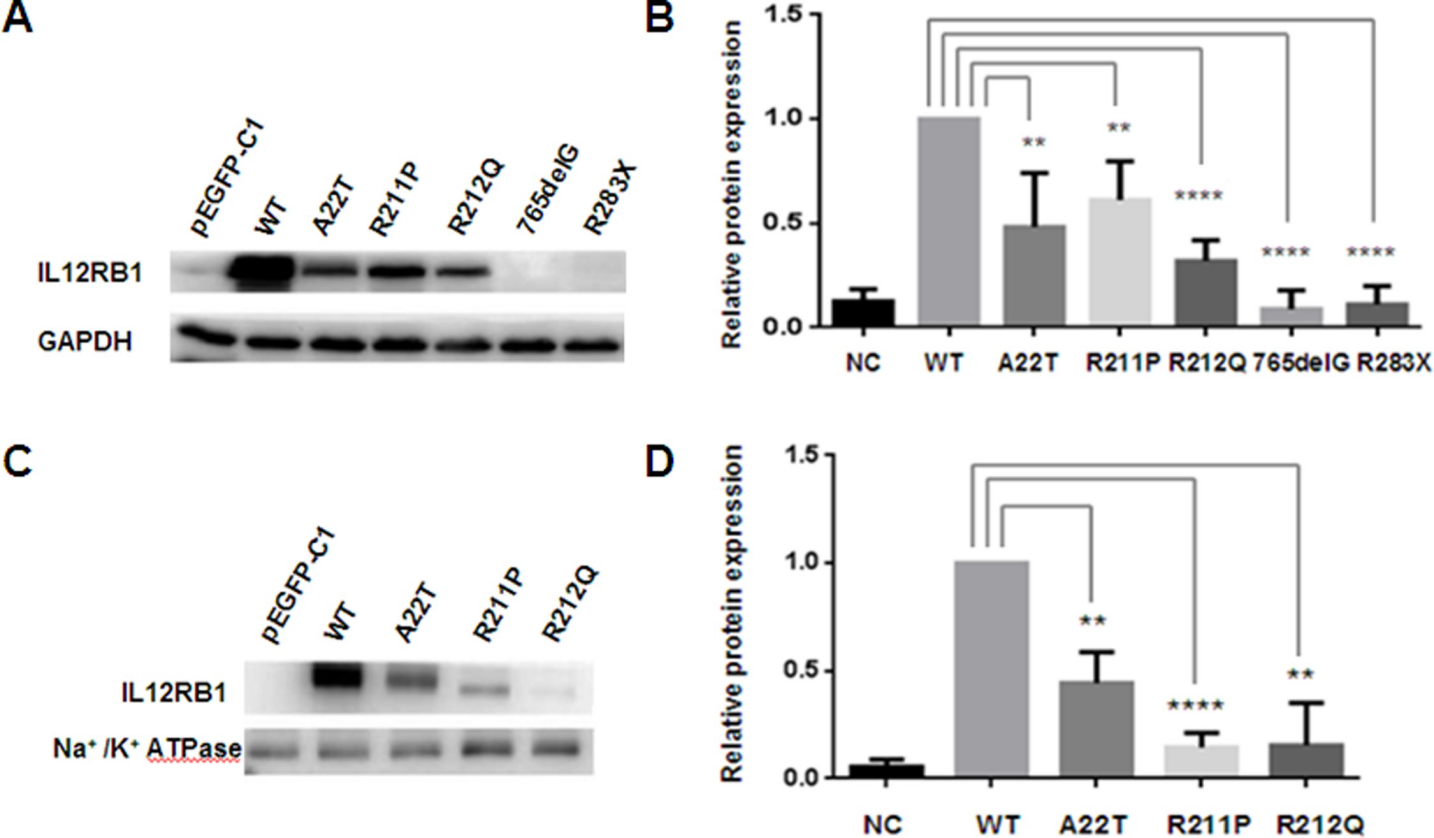
Western blot analysis of wild type and mutant IL-12Rβ1 proteins. **4A)** The expression level of IL-12Rβ1. **4B)** Relative total protein expression of wild and mutant IL-12Rβ1(A22T, R211P, R212Q, 765delG, R283X). **4C)** The expression level of IL-12Rβ1 in cell membrane. Missense mutations (A22T, R211P, R212Q) lead to reduce expression level of IL-12Rβ1 in the cell membrane. **4D)** Relative expression level of wild and mutant (A22T, R211P, R212Q) IL-12Rβ1 in cell membrane. Results represent the average ± SEM of three independent experiments (****p< 0.0001, **p< 0.01, n>3).

We checked the protein expression of wild type and mutant *IL12RB1* gene in HEK293T cells. HEK293T cells were transfected with plasmids of pEGFP-C1-WT IL12Rβ1 and pEGFP-C1-mutant IL12Rβ1(A22T, R211P, R212Q, 765delG, R283X) respectively. Missense mutations (A22T, R211P, R212Q) lead to reduce the IL12RB1 expression level in different degree, while nonsense mutation (R283X) and frameshift mutation(765delG) result in degradation of IL-12Rβ1 protein (Fig 4A and 4B).

### Expression level of IL-12Rβ1 in cell membrane of HEK293T cells

Subcellular distribution indicated wild type IL-12Rβ1 protein was detected mainly localized in the plasma membrane, while mutant IL-12Rβ1 protein mainly localized in the cytoplasm, so we check the expression level of wild type and mutant IL12 Rβ1 proteins in the cell membrane in HEK293T cells. These missense mutations (A22T, R211P, R212Q) lead to reduce the expression of IL-12Rβ1 in the cell membrane (Fig 4C and 4D).

## Discussion

BCG vaccine is the most commonly used to newborns. It is considered to be safe, but some complications have been reported like cellulitis, abscesses at the site of inoculation, regional lymphadenitis (BCGitis) and disseminated BCG infection (BCG-osis) [11]. Futhermore, the vast majority of the patients with complications reported with MSMD. The infections with the live attenuated Mycobacterium bovis Bacille Calmette-Guerin strain (BCG) of the tuberculosis (TB) vaccine, usually occurred only once in these patients [12].

Since first disease gene of MSMD was reported in 1996 [2], mutations in eleven disease genes of MSMD have been reported [1, 3, 4]. Currently, in China, almost every child will receive BCG vaccine after birth to prevent tuberculosis. The genetic causes of Chinese with MSMD which had been reported including *IL12RB1*[10] and *IFNGR1*[13]. Few relevant data indicate there is insufficient understanding of the disease in China at present. The genetic study of MSMD has important immunological and clinical implications. At present, most patients with MSMD are treated with antibiotics, with or without recombinant IFN-γ, severe patients are treated with hematopoietic stem cell transplantation (HSCT) [1].

In this study, we identified three novel compound heterozygous mutations in *IL12RB1* gene (c.635G > A, c.765delG; c.632G > C, c.847C > T; c.64G > A, c.1673insGAGCTTCCTGAG) in three unrelated patients with MSMD. One of the heterozygous mutation c.632G > C has been discovered in a Taiwanese patient (with MSMD) who carries the homozygous mutation [13] and heterozygous mutation c.847C > T has been reported in an Iranian patient (with MSMD) who also carries the homozygous mutation [14]. Here, we identified each patient carries two heterozygous mutations, and one came from his/her mother while the other came from his/her father. Moreover, their parents did not suffer from MSMD and 100 unrelated normal controls who received BCG vaccine and without MSMD did not carry these mutations. Subcellular localization analysis show that missense mutations affect the traffic of IL-12Rβ1 protein in the cell. Furthermore, Western blot analysis show that mutations reduce the expression level of IL-12Rβ1 total protein or expression level on cell membrane. IL-12Rβ1 is the receptor of IL12 signaling pathway to produce IFN-γ, loss function of mutations have been reported to reduce the IFN-γ production and will cause the infection of BCG, we speculate that the loss of function mutations which we identified will also will affected the IL12 signaling pathway and IFN-γ production. Our study not only enriches the *IL12RB1* mutation database, but also provides patients with a more accurate diagnosis as well as precise treatment choices of Chinese MSMD patients.
